# Supplementary material for: Macroscale and microcircuit dissociation of focal and generalized human epilepsies
Source: Commun Biol. 2020 May 18;3:244. doi: 10.1038/s42003-020-0958-5 (PMC7234993; doi:10.1038/s42003-020-0958-5)
Supplement: Supplementary file 1 — Supplementary Information [file 42003_2020_958_MOESM1_ESM.pdf]

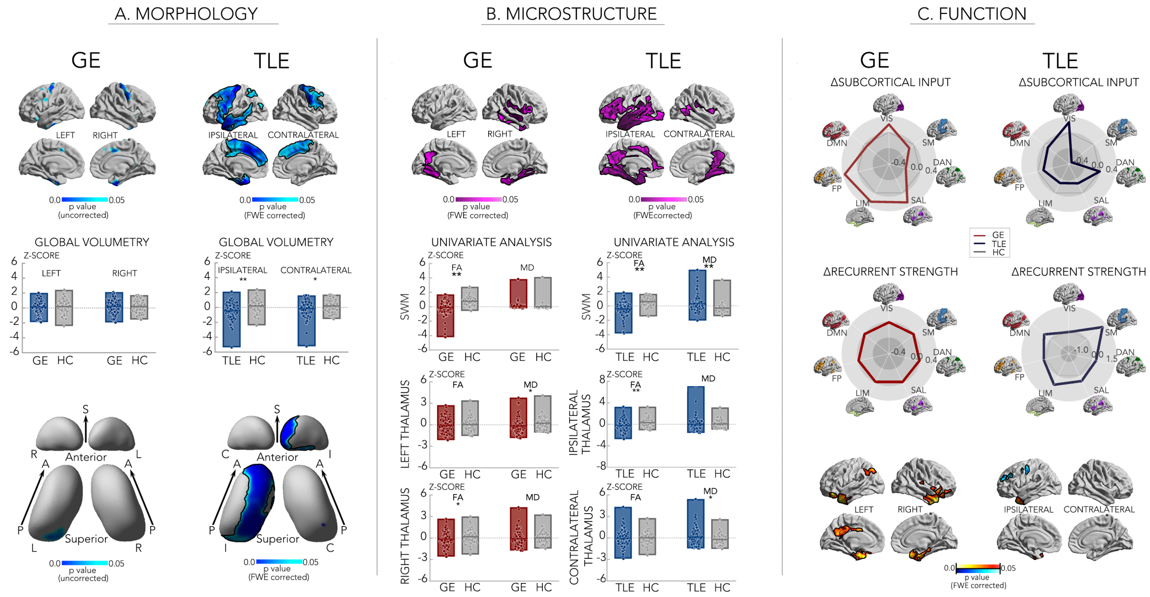

Supplementary Figure 1 | Comparison between individual patient groups (107 TLE, temporal lobe epilepsy; 96 GE, idiopathic/genetic generalized epilepsy) and 65 healthy controls (HC). ***A***. Morphological analysis. ***B.*** Microstructural analysis. ***C.*** Functional and connectome-informed model parameter alterations. Surface-based findings were corrected for multiple comparisons at a family-wise level of 0.05 (*black outlines*), trends are shown in semi-transparent. Stars indicate significances of thalamic and subcortical input findings (**p*<0.05; ***p*<0.01). *Note:* L, left; R, right; S, superior; A, anterior; P, posterior; C, contralateral; I: ipsilateral.

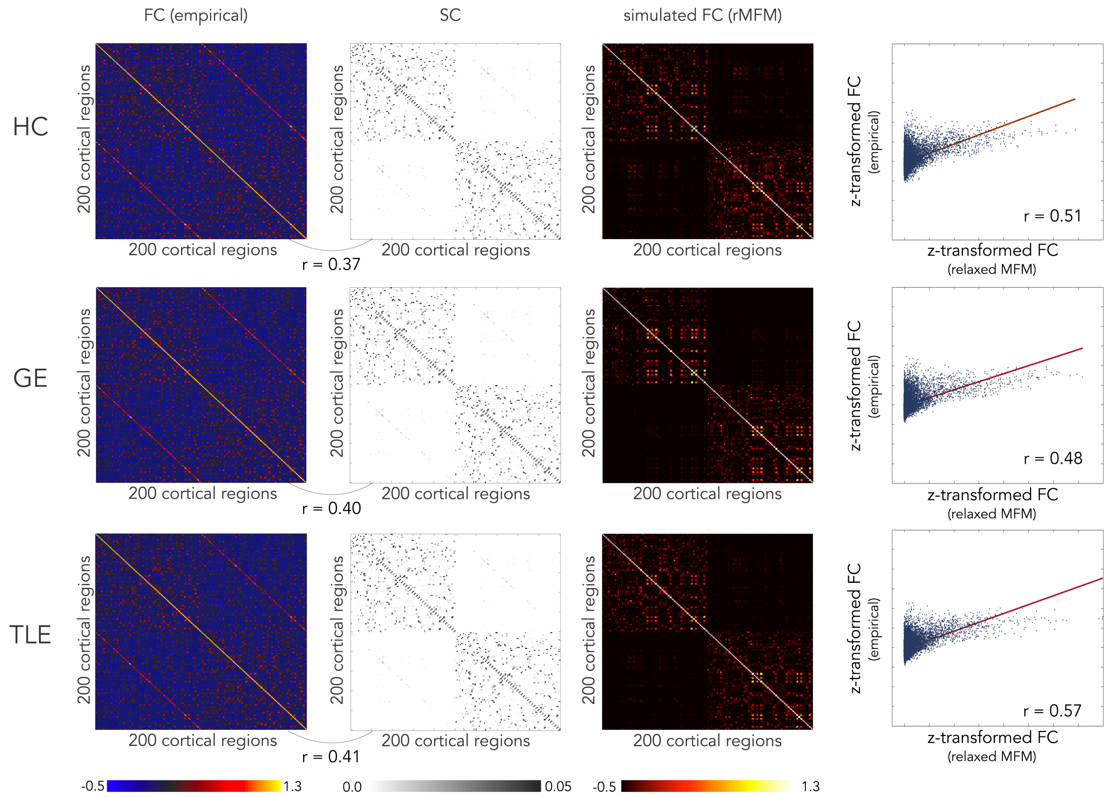

Supplementary Figure 2 | Correspondence between empirical and simulated functional connectivity. The 200×200 empirical global functional connectivity (FC), structural connectivity (SC), and simulated FC based on the relaxed mean field model (rMFM). Correlations between SC and empirical FC were 0.37, 0.40 and 0.41 in HC, GE, and TLE. Correlations between simulated and empirical FC were 0.51, 0.48 and 0.57 in HC, GE, and TLE.

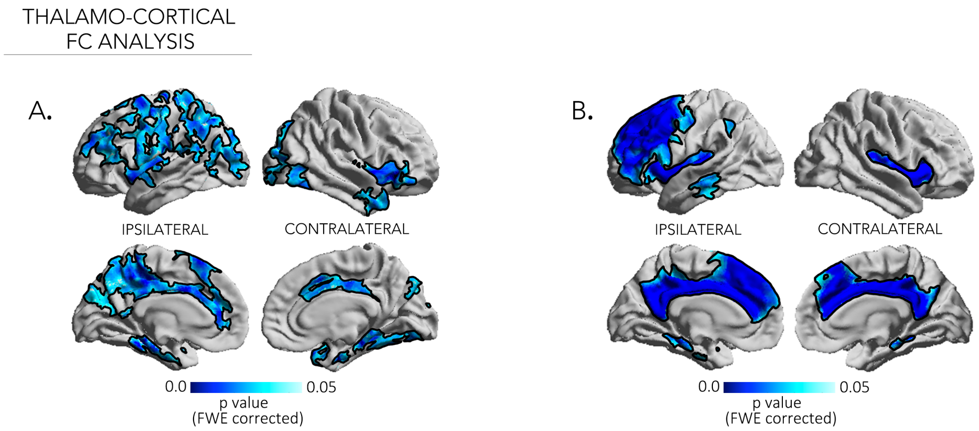

Supplementary Figure 3 | Thalamo-cortical functional connectivity differences between 107 TLE and 96 GE patients with (B) /without (A) additionally regressing out the global mean signal. Surface-based findings were corrected for multiple comparisons at a family-wise level of 0.05 (*black outlines*)

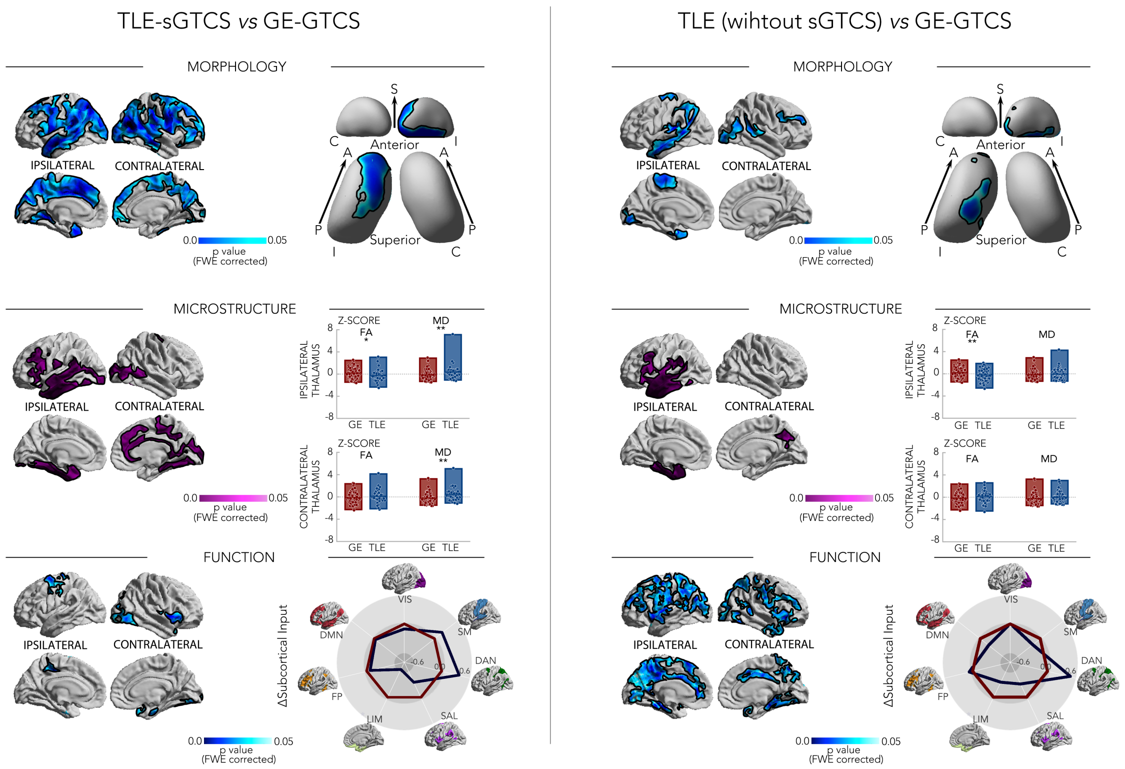

SUPPLEMENTARY FIGURE 4 | Between cohort-differences when separately restricting the TLE cohort to those with/without secondary generalized tonic clonic seizures. Surface-based findings (*i.e.,* cortical thickness, SWM, and thalamo-cortical connectivity) were corrected for multiple comparisons at a family-wise level of 0.05 (black outline), trends are shown in semi-transparent. Stars indicate significances of thalamic findings ($^{*}p<0.05$; $^{**}p<0.01$). *Note:* S, superior; A, anterior; P, posterior; C, contralateral; I: ipsilateral.

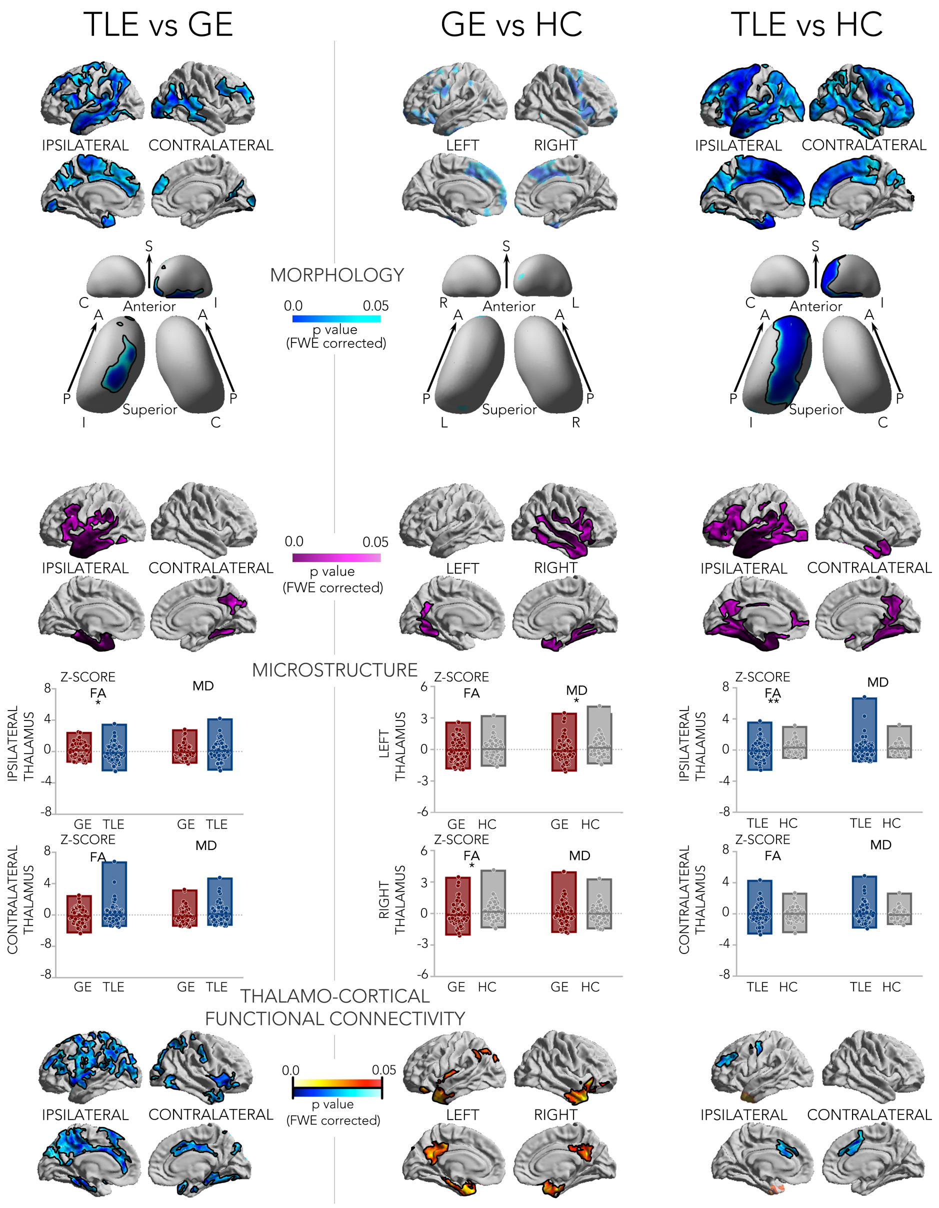

Supplementary Figure 5 | Between cohort-differences after controlling for drug-response. Surface-based findings (*i.e.,* cortical thickness, superficial white matter microstructure, and thalamo-cortical connectivity) were corrected for multiple comparisons at a family-wise level of 0.05 (black outline), trends are shown in semi-transparent. Stars indicate significances of thalamic findings (*p<0.05; **p<0.01). *Note:* L, left; R, right; S, superior; A, anterior; P, posterior; C, contralateral; I: ipsilateral.

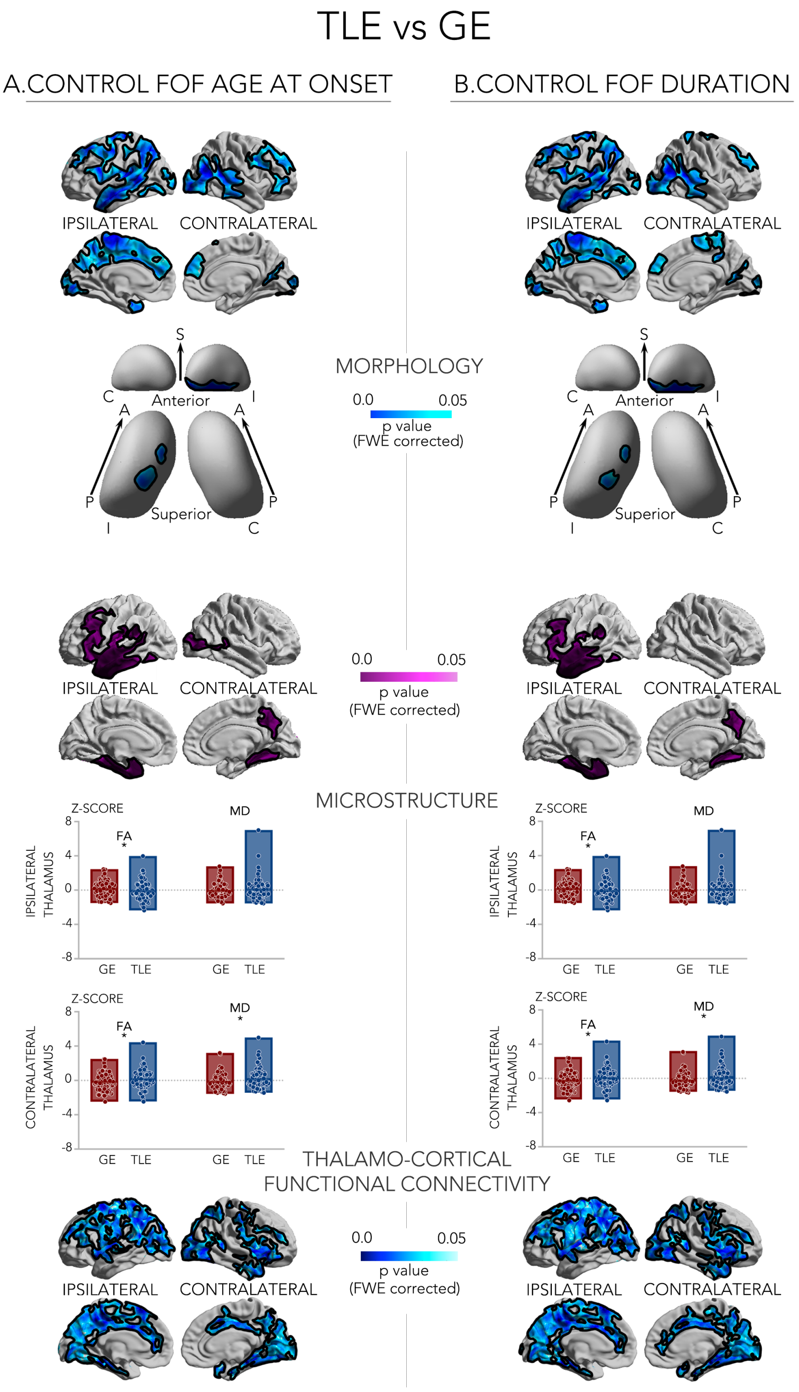

Supplementary Figure 6 | Between cohort-differences after controlling for age at seizure onset and duration of epilepsy. Surface-based findings (*i.e.,* cortical thickness, superficial white matter microstructure, and thalamo-cortical connectivity) were corrected for multiple comparisons at a family-wise level of 0.05 (black outline), trends are shown in semi-transparent. Stars indicate significances of thalamic findings ($^{*}p<0.05$; $^{**}p<0.01$). *Note:* L, left; R, right; S, superior; A, anterior; P, posterior; C, contralateral; I: ipsilateral.

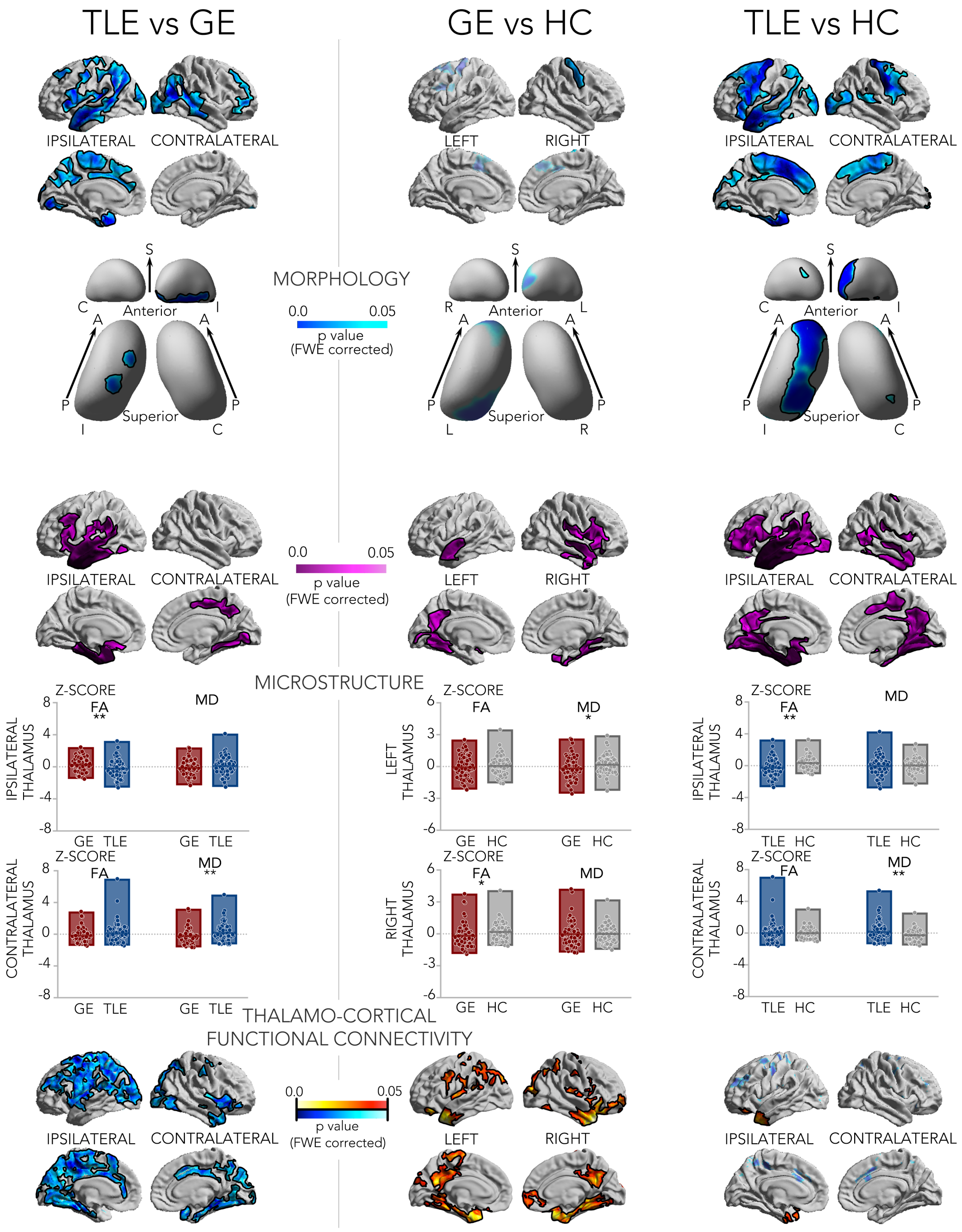

Supplementary Figure 7 | Between cohort-differences when restricting the sample to patients who had at least one seizure/year and at least one year of epilepsy duration. Surface-based findings (*i.e.,* cortical thickness, superficial white matter microstructure, and thalamo-cortical connectivity) were corrected for multiple comparisons at a family-wise level of 0.05 (black outline), trends are shown in semi-transparent. Stars indicate significances of thalamic findings ($^{*}p<0.05$; $^{**}p<0.01$). *Note:* L, left; R, right; S, superior; A, anterior; P, posterior; C, contralateral; I: ipsilateral.

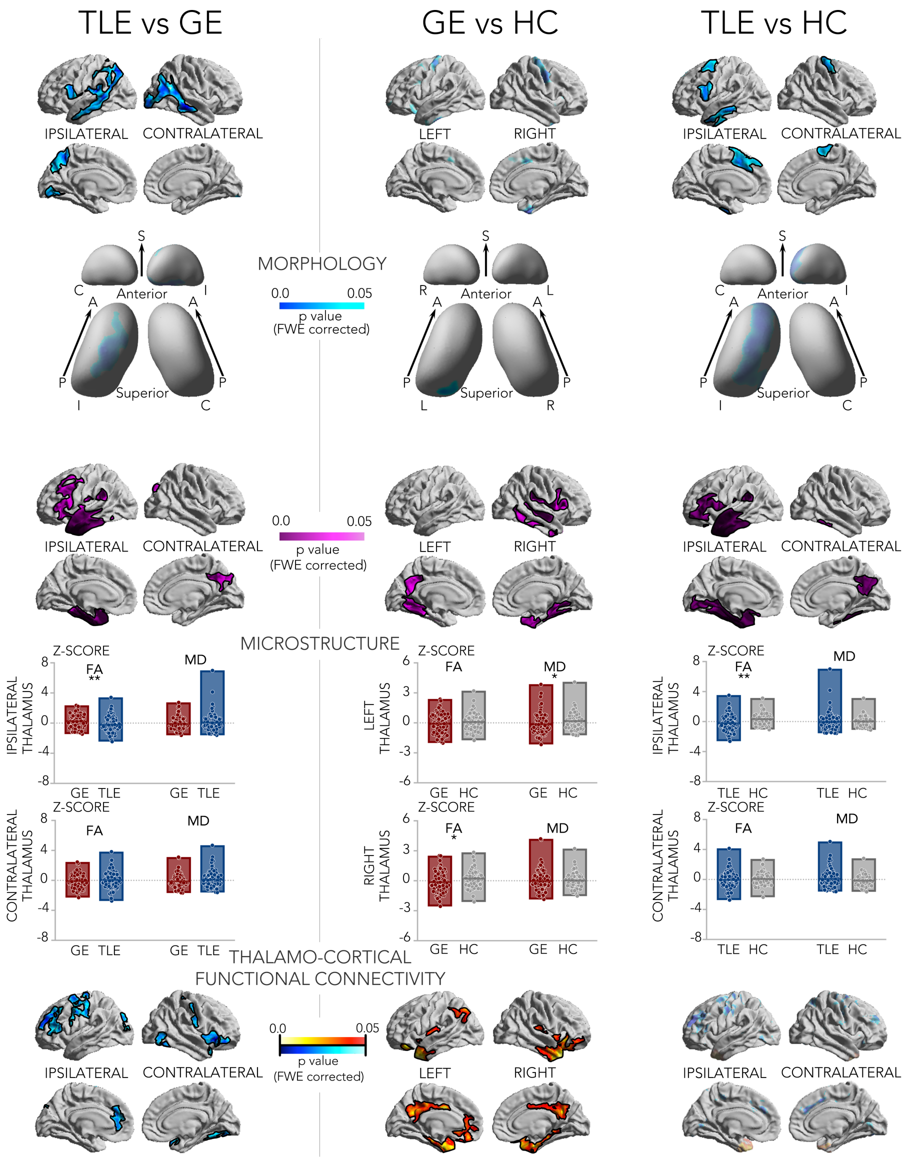

Supplementary Figure 8 | Between cohort-differences after controlling for hippocampal volume. Surface-based findings (*i.e.,* cortical thickness, superficial white matter microstructure, and thalamo-cortical connectivity) were corrected for multiple comparisons at a family-wise level of 0.05 (black outline), trends are shown in semi-transparent. Stars indicate significances of thalamic findings ($^{*}p<0.05$; $^{**}p<0.01$). *Note:* L, left; R, right; S, superior; A, anterior; P, posterior; C, contralateral; I: ipsilateral.

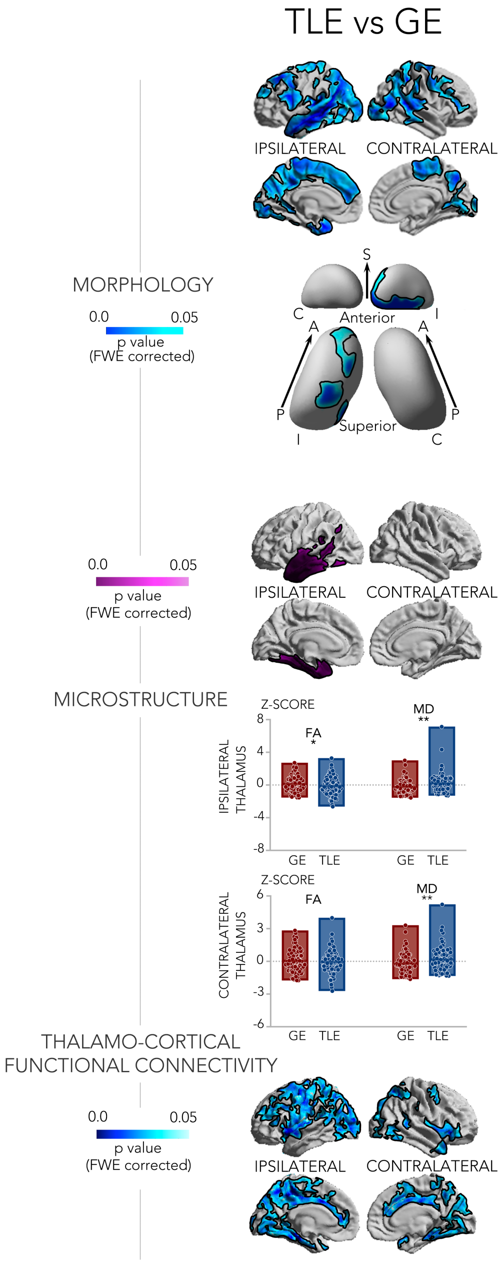

SUPPLEMENTARY FIGURE 9 | Between cohort-differences after also left-right flipping a similar proportion of GE patients as the controls. Of note, measures in patients were z-scored relative to corresponding measures in controls prior to flipping. Surface-based findings (*i.e.,* cortical thickness, superficial white matter microstructure, and thalamo-cortical connectivity) were corrected for multiple comparisons at a family-wise level of 0.05 (black outline), trends are shown in semi-transparent. Stars indicate significances of thalamic findings ($^{*}p<0.05$; $^{**}p<0.01$). *Note:* L, left; R, right; S, superior; A, anterior; P, posterior; C, contralateral; I: ipsilateral.
