# Supplementary material for: Macroscale and microcircuit dissociation of focal and generalized human epilepsies
Source: Commun Biol. 2020 May 18;3:244. doi: 10.1038/s42003-020-0958-5 (PMC7234993; doi:10.1038/s42003-020-0958-5)
Supplement: Supplementary file 2 — Reporting Summary [file 42003_2020_958_MOESM2_ESM.pdf]

nature research

Corresponding author(s): Boris Berhardt, Zhiqiang Zhang

Last updated by author(s): Mar 23, 2020

Reporting Summary

Nature Research wishes to improve the reproducibility of the work that we publish. This form provides structure for consistency and transparency in reporting. For further information on Nature Research policies, see Authors & Referees and the Editorial Policy Checklist.

Statistics

For all statistical analyses, confirm that the following items are present in the figure legend, table legend, main text, or Methods section.

n/a	Confirmed	
☐	☒	The exact sample size (*n*) for each experimental group/condition, given as a discrete number and unit of measurement
☐	☒	A statement on whether measurements were taken from distinct samples or whether the same sample was measured repeatedly
☐	☒	The statistical test(s) used AND whether they are one- or two-sided<br>*Only common tests should be described solely by name; describe more complex techniques in the Methods section.*
☐	☒	A description of all covariates tested
☐	☒	A description of any assumptions or corrections, such as tests of normality and adjustment for multiple comparisons
☐	☒	A full description of the statistical parameters including central tendency (e.g. means) or other basic estimates (e.g. regression coefficient) AND variation (e.g. standard deviation) or associated estimates of uncertainty (e.g. confidence intervals)
☐	☒	For null hypothesis testing, the test statistic (e.g. *F*, *t*, *r*) with confidence intervals, effect sizes, degrees of freedom and *P* value noted<br>*Give P values as exact values whenever suitable.*
☒	☐	For Bayesian analysis, information on the choice of priors and Markov chain Monte Carlo settings
☒	☐	For hierarchical and complex designs, identification of the appropriate level for tests and full reporting of outcomes
☐	☐	Estimates of effect sizes (e.g. Cohen's *d*, Pearson's *r*), indicating how they were calculated

Our web collection on statistics for biologists contains articles on many of the points above.

Software and code

Policy information about availability of computer code

Data collection	Statistical procedures from the SurfStat toolbox, together with documentation, are openly accessible via http://www.math.mcgill.ca/keith/surfstat and http://mica-mni.github.io/surfstat.
Data analysis	All statistical analyses analyses were carried out using SurfStat for Matlab;

For manuscripts utilizing custom algorithms or software that are central to the research but not yet described in published literature, software must be made available to editors/reviewers. We strongly encourage code deposition in a community repository (e.g. GitHub). See the Nature Research guidelines for submitting code & software for further information.

Data

Policy information about availability of data

All manuscripts must include a data availability statement. This statement should provide the following information, where applicable:

- Accession codes, unique identifiers, or web links for publicly available datasets
- A list of figures that have associated raw data
- A description of any restrictions on data availability

Feature data are available via osf.io (doi: 10.17605/OSF.IO/GQXES)

Field-specific reporting

Please select the one below that is the best fit for your research. If you are not sure, read the appropriate sections before making your selection.

☒ Life sciences ☐ Behavioural & social sciences ☐ Ecological, evolutionary & environmental sciences

Life sciences study design

All studies must disclose on these points even when the disclosure is negative.

Sample size	In brief, we studied 107 TLE patients with unilateral hippocampal atrophy, 96 GE patients with generalized tonic clonic seizures as their only seizure type, and 65 healthy controls. Patient cohorts had a comparable age and sex distribution, and underwent identical 3T multimodal MRI.
Data exclusions	We studied 263 epilepsy patients recruited from Jinling Hospital, Nanjing, China between July 2009 and August 2018. Patients were diagnosed as having either GE with generalized tonic-clonic seizures or unilateral TLE with MRI evidence for hippocampal sclerosis. Diagnoses followed ILAE criteria 65, and were informed by electro-clinical factors, neurological examination, and neuroimaging. Further inclusion criteria were: (i) age older than 16 years; (ii) right-handedness; (iii) no mass lesion (i.e., brain tumor, cerebral haemorrhage or ischemia, cerebrovascular malformation); (iv) no history of brain surgery; (v) no significant physical conditions; (vi) no alcohol or substance abuse; (vii) no MRI contraindications. Among the initial 263 patients, we selected only those with available MRI data for all the studied modalities and those who did not present with imaging artifacts. Our final patient cohort consisted of 203 patients: 96 GE (31 females, mean±SD age=25.65±7.85 years) and 107 TLE patients (53 left and 54 right TLE; 47 females, mean±SD age=27.29±7.81 years). Patients were compared to 65 age- and sex-matched healthy controls (HCs; 25 females, mean±SD age=24.98±4.96 years).
Replication	Several sensitivity analyses assessed robustness and consistency of our main findings.
Randomization	Case-control study that compared patient groups (TLE, GE) to healthy controls.
Blinding	Multimodal image processing was done using automated pipelines.

Reporting for specific materials, systems and methods

We require information from authors about some types of materials, experimental systems and methods used in many studies. Here, indicate whether each material, system or method listed is relevant to your study. If you are not sure if a list item applies to your research, read the appropriate section before selecting a response.

Materials & experimental systems

n/a	Involved in the study	
☒	☐	Antibodies
☒	☐	Eukaryotic cell lines
☒	☐	Palaeontology
☒	☐	Animals and other organisms
☐	☒	Human research participants
☒	☐	Clinical data

Methods

n/a	Involved in the study	
☒	☐	ChIP-seq
☒	☐	Flow cytometry
☐	☒	MRI-based neuroimaging

Human research participants

Policy information about studies involving human research participants

Population characteristics	Detailed socio-demographic and clinical information can be found in TABLE 1.
Recruitment	Patients were recruited from Jinling Hospital, Nanjing, China between July 2009 and August 2018.
Ethics oversight	This study was carried out according to the declaration of Helsinki and approved by the ethics committee of Jinling Hospital.

Note that full information on the approval of the study protocol must also be provided in the manuscript.

Magnetic resonance imaging

Experimental design

Design type	na
Design specifications	na
Behavioral performance measures	na

Acquisition

Imaging type(s): Structural, resting-state functional, diffusion

Field strength: 3 Tesla

Sequence & imaging parameters: Data were acquired on a 3T MRI scanner (TIM Trio, Siemens Medical Solution, Erlangen, Germany) equipped with an 8-channel head coil. We used a 3D rapid gradient echo sequence to acquire high-resolution T1-weighted MRI (T1w; 176 slices; repetition time [TR] = 2300 ms; echo time [TE] = 2.98 ms; flip angle = 9°; field of view [FOV] = 256 × 256 mm2; 0.5 × 0.5 × 1 mm3 voxels) and a 2D echo-planar imaging spin echo sequence to acquire diffusion MRI (DWI; 45 slices; TR = 6100ms; TE = 93 ms; 120 volumes with non-collinear directions [b = 1000 s/mm2] and 4 volumes without diffusion weighting [b = 0 s/mm2]; FOV = 240 × 240 mm2; 0.94 × 0.94 × 3 mm3 voxels). Using 2D echo-planar BOLD imaging, we acquired resting-state fMRI (rs-fMRI; 30 slices; TR = 2000ms; TE = 30 ms; flip angle, 90°; FOV = 240 × 240 mm2; 250 volumes; 3.75 × 3.75 × 4 mm3 voxels).

Area of acquisition: *State whether a whole brain scan was used OR define the area of acquisition, describing how the region was determined.*

Diffusion MRI: ☒ Used ☐ Not used

Parameters: 120 directions, b=1000, single shell

Preprocessing

Preprocessing software: We processed T1w data using FreeSurfer (v6.0; http://surfer.nmr.mgh.harvard.edu/) to generate models of the cortical surface and to index neocortical morphology. The entire thalamus was automatically segmented using FSL-FIRST (v5.0.9; https://fsl.fmrib.ox.ac.uk/fsl/fslwiki/FIRST/). Diffusion MRI data were preprocessed with MRtrix (v0.3.15; http://www.mrtrix.org/).The rs-fMRI processing was conducted via DPARSF (v2.3; http://www.rfmri.org/DPARSF).

Normalization: Cortical surface measures underwent surface-based registration via FreeSurfer. Diffusion and fMRI data were mapped to surfaces via boundary based registrations and then registered to the Conte69 template. Thalamic segmentations were linearly registered to MNI152.

Normalization template: Conte69, MNI152

Noise and artifact removal: FreeSurfer based processing involves non-uniformity correction. Diffusion MRI processing included included head motion and eddy current correction, de-noising, as well as diffusion parameter estimation. For the rs-fMRI data, the first 10 images were excluded to ensure steady-state signal equilibrium. Images underwent correction for slice timing, realignment, band-pass filtering (0.01-0.1Hz), and spatial smoothing using a 6mm full-width-at-half-maximum Gaussian kernel. We statistically corrected for head motion as well as average white matter and cerebrospinal fluid signals.

Volume censoring: DPARSF for the fMRI analysis

Statistical modeling & inference

Model type and settings: Linear models implemented in SurfStat

Effect(s) tested: Between-group differences

Specify type of analysis: ☐ Whole brain ☐ ROI-based ☒ Both

Anatomical location(s): Thalamus and neocortex

Statistic type for inference (See Eklund et al. 2016): Cluster level

Correction: FWE

Models & analysis

n/a	Involved in the study
☐	☒ Functional and/or effective connectivity
☒	☐ Graph analysis
☒	☐ Multivariate modeling or predictive analysis

Functional and/or effective connectivity: Pearson correlation
